# Tuberculosis-Associated Scar Carcinoma in Lung Cancer: Clinicopathological and Radiological Features of a Fibrotic-Cavitary Phenotype in a Retrospective Observational Cohort

**DOI:** 10.3390/cancers18121935

**Published:** 2026-06-14

**Authors:** Cristina Cioti, Irina Tica, Cristina Tocia, Nejla Dervis, Simona Buligan, Gabriela Fricatel, Denisa Gabriela Ion-Andrei, Oana Cristina Arghir

**Affiliations:** 1 Internal Medicine Department, “Sf. Apostol Andrei” Emergency County Hospital, 145 Tomis Blvd., 900591 Constanta, Romania; cristina.cioti@365.univ-ovidius.ro (C.C.); irina.tica@univ-ovidius.ro (I.T.); 2 PhD School of Medicine, “Ovidius” University of Constanta, 1 University Street, 900470 Constanta, Romania; denisa.ia@365.univ-ovidius.ro (D.G.I.-A.); arghir_oana@yahoo.com (O.C.A.); 3 Gastroenterology Department, “Sf. Apostol Andrei” Emergency County Hospital, 145 Tomis Blvd., 900591 Constanta, Romania; simona.culacsiz@drd.umfcd.ro; 4 Medicine Faculty, “Ovidius” University of Constanta, 1 Universitatii Street, 900470 Constanta, Romania; nejla.dervis@365.univ-ovidius.ro; 5 PhD School of Medicine, Carol Davila University of Medicine and Pharmacy, 8 Eroii Sanitari Boulevard, 050474 Bucharest, Romania; 6 Oncology Department, “Sf. Apostol Andrei” Emergency County Hospital, 145 Tomis Blvd., 900591 Constanta, Romania; 7 Clinical Pneumology Hospital of Constanta, 40 Sentinelei Str, 900002 Constanta, Romania

**Keywords:** scar carcinoma, pulmonary tuberculosis, lung cancer, radiological overlap, cavitary lesions, pulmonary imaging, post-tuberculosis sequelae

## Abstract

Background: Scar carcinoma represents a distinct subtype of lung malignancy developing in areas of chronic pulmonary fibrosis, inflammation, and structural remodeling, frequently associated with previous pulmonary tuberculosis (TB). The present study aimed to evaluate the radiological, clinical, inflammatory, and histopathological characteristics associated with the scar carcinoma phenotype in patients with lung cancer (LC) and previous TB-related pulmonary abnormalities. Methods: A retrospective observational cohort study conducted between February 2020 and December 2025 included 844 patients diagnosed with lung cancer. The scar carcinoma phenotype was operationally defined by the coexistence of: (1) confirmed lung cancer, (2) post-tuberculous structural pulmonary abnormalities on thoracic imaging, and (3) clinical history compatible with prior pulmonary TB. Associations between the scar carcinoma phenotype and clinicopathological variables were evaluated using Pearson’s Chi-square and Fisher’s exact tests. Binary logistic regression analysis was performed to identify independent predictive factors associated with scar carcinoma. Receiver operating characteristic (ROC) curve and precision–recall curve analyses were additionally performed. Results: Post-TB sequelae were identified in 58.2% of patients, while active TB was present in 7.8% of cases. Adenocarcinoma represented the predominant histopathological subtype (63.3%). Fibrotic/interstitial/bronchial abnormalities (67.7%), cavitary/destructive lesions (69.0%), atelectatic/retractile changes (65.4%), and infectious/inflammatory pulmonary abnormalities (60.4%) were highly prevalent. Significant associations were identified between scar carcinoma and TB sequelae (χ^2^ = 811.850, *p* < 0.001), adenocarcinoma histology (χ^2^ = 655.545, *p* < 0.001), infectious/inflammatory changes (χ^2^ = 635.168, *p* < 0.001), cavitary/destructive lesions (χ^2^ = 508.347, *p* < 0.001), fibrotic/interstitial abnormalities (χ^2^ = 539.895, *p* < 0.001), and atelectatic/retractile changes (χ^2^ = 597.346, *p* < 0.001). Logistic regression identified haemoptysis (OR = 0.651 (95% CI: 0.486–0.871), *p* = 0.005) and pulmonary opacities and/or condensation (OR = 1.343 (95% CI: 1.014–1.779), *p* = 0.040) as independent predictive factors. ROC analysis demonstrated moderate predictive performance (AUC = 0.703). Conclusions: TB-associated pulmonary remodeling was strongly associated with the scar carcinoma phenotype, consistent with an associative role of chronic inflammation, fibrosis, and post-TB structural damage in lung carcinogenesis; however, causal inferences cannot be drawn from this retrospective observational design.

## Simple Summary

Pulmonary tuberculosis leaves permanent scars in the lung tissue even after the infection has been treated. In some patients, these areas of chronic scarring and inflammation may later give rise to a type of lung cancer known as scar carcinoma. However, the clinical, radiological, and pathological characteristics of this cancer type remain poorly described, partly because no universally accepted criteria for its diagnosis exist. This study analyzed 844 patients with lung cancer who also had signs of previous tuberculosis infection, with the aim of identifying the radiological patterns, symptoms, and tissue findings most strongly linked to the scar carcinoma profile. We found that structural lung damage was closely associated with this cancer pattern. These findings may help clinicians recognize high-risk patients earlier and support the development of better surveillance strategies for people with a history of pulmonary tuberculosis.

## 1. Introduction

Scar carcinoma represents a distinct subtype of lung malignancy developing in areas of previous pulmonary fibrosis, chronic inflammation, and structural scarring [1]. The concept was first introduced in the early twentieth century to describe carcinomas arising adjacent to fibrotic pulmonary lesions, particularly those associated with healed pulmonary tuberculosis [2]. Among the multiple etiologies of pulmonary scarring, post-tuberculous fibrotic remodeling remains the most frequently implicated substrate for scar-associated carcinogenesis [3]. Recent evidence has reinforced the association between previous pulmonary tuberculosis and subsequent lung cancer development, especially in patients presenting chronic inflammatory pulmonary damage, cavitary lesions, and architectural distortion [4,5].

Tuberculosis continues to represent a major global health burden and remains one of the leading infectious causes of mortality worldwide [6]. Simultaneously, lung cancer is the most commonly diagnosed malignancy and the leading cause of cancer-related death globally. Increasing epidemiological evidence has demonstrated that individuals with previous pulmonary tuberculosis exhibit a significantly increased risk of developing lung cancer compared to the general population, independent of smoking and other classical risk factors [7]. Several cohort studies have reported a two- to threefold increase in lung cancer incidence among patients with prior TB infection [8,9].

The biological relationship between tuberculosis and lung carcinogenesis appears to be multifactorial. Persistent inflammatory stimulation induced by *Mycobacterium tuberculosis* infection promotes repeated cycles of tissue injury and repair, ultimately leading to fibrosis and scar formation [10]. Chronic inflammatory microenvironments are characterized by increased production of reactive oxygen species, inflammatory cytokines, tumor necrosis factor-α, interleukin-1, interleukin-6, cyclooxygenase-2, and nuclear factor-kappa B, all of which contribute to DNA damage, genomic instability, impaired apoptosis, and abnormal cellular proliferation [11]. Furthermore, chronic fibrotic remodeling and cavitary destruction may alter the pulmonary microarchitecture and create a favorable milieu for malignant transformation [12].

**Figure 1:**
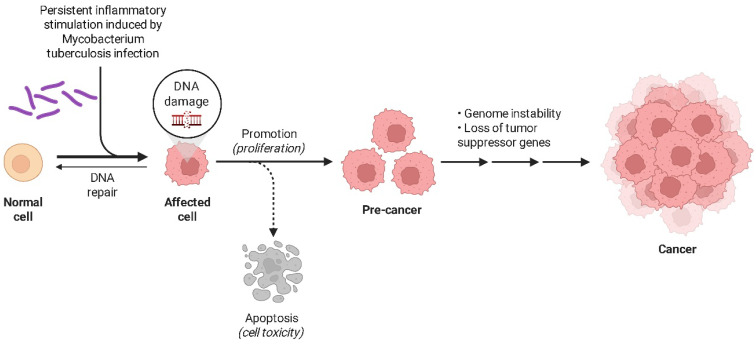
Proposed mechanism of tuberculosis-associated scar carcinogenesis through chronic inflammatory injury and genomic instability. Created in BioRender. Cristina, C. (2026) https://BioRender.com/x44lq4t (accessed on 11 March 2026) [13].

Figure 1 illustrates the proposed biological mechanisms linking chronic post-tuberculous inflammation, fibrosis, genomic instability, and malignant transformation involved in scar-associated carcinogenesis.

**Figure 2:**
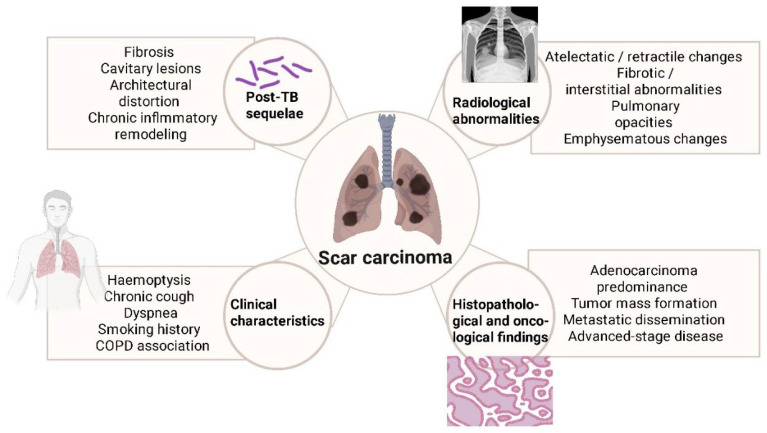
Clinicopathological and radiological characteristics associated with the scar carcinoma phenotype. Created in BioRender. Cristina, C. (2026) https://BioRender.com/x9tzpze (accessed on 16 March 2026) [13].

The complex relationship between previous pulmonary tuberculosis, chronic inflammatory remodeling, and malignant transformation is summarized in Figure 2. The proposed scar carcinoma phenotype is characterized by the coexistence of post-tuberculous structural abnormalities, radiological fibrotic and cavitary changes, persistent inflammatory pulmonary injury, and distinct clinicopathological features.

Several histopathological subtypes have been associated with scar carcinoma, although adenocarcinoma is most frequently reported in the literature [14]. Scar-associated tumors are often located in the upper pulmonary lobes and may coexist with cavitary lesions, fibrotic bands, bronchiectatic distortion, or chronic inflammatory infiltrates [15]. In clinical practice, differentiating scar carcinoma from residual post-tuberculous abnormalities remains challenging due to overlapping radiological features. Consequently, delayed diagnosis and advanced-stage presentation are common among affected patients [16].

Despite increasing recognition of the relationship between tuberculosis and lung cancer, the clinicopathological profile of scar carcinoma remains insufficiently characterized [17]. Most currently available data originate from retrospective studies, case reports, or narrative reviews, while standardized diagnostic criteria remain limited. Therefore, further investigation is required to better define the radiological, clinical, and pathological characteristics associated with scar carcinoma development in patients with previous pulmonary tuberculosis [18].

In the present study, the scar carcinoma phenotype was operationally defined through a composite of three concurrent criteria: (1) a confirmed diagnosis of LC established through histopathological, radiological, or multidisciplinary clinical evaluation; (2) the presence of post-TB structural pulmonary abnormalities identified on thoracic imaging, including fibrotic and interstitial changes, cavitary or destructive lesions, and atelectatic or retractile abnormalities; and (3) a clinical history compatible with prior pulmonary TB, based on documented previous diagnosis, prior anti-tuberculous treatment, or characteristic imaging findings in the absence of alternative explanations. This composite definition was subsequently translated into categorical variables and analyzed using Pearson’s chi-square and Fisher’s exact tests to assess associations with clinicopathological and radiological features, and by binary logistic regression analysis to identify independent predictors of the scar carcinoma phenotype within the study cohort.

Despite increasing recognition, no universally accepted operational definition of scar carcinoma exists, and most available evidence derives from case reports or narrative reviews. We also addressed this gap by providing a large-cohort, multivariate characterization of the scar carcinoma phenotype in a population with high TB prevalence.

Our main aim was to evaluate the clinicopathological, radiological, and inflammatory characteristics associated with the scar carcinoma phenotype in this cohort of patients with lung cancer and previous tuberculosis-related pulmonary abnormalities. Additionally, the study aimed to investigate the association between post-tuberculous structural remodelling, chronic inflammatory pulmonary changes, and malignant transformation through both univariate and multivariate statistical analyses.

## 2. Materials and Methods

### 2.1. Study Design and Setting

This retrospective observational study was conducted over a six-year period, between February 2020 and December 2025 in Clinical Pneumophtisiology Hospital of Constanța. The study focused on patients diagnosed with lung cancer and aimed to investigate the clinical and radiological overlap between pulmonary tuberculosis and lung malignancy, with emphasis on tuberculosis status, imaging characteristics, respiratory manifestations, and oncological staging.

### 2.2. Study Population

During the study period, all hospitalized patients with documented pulmonary malignancy were screened for eligibility. Patients were included if they had a confirmed diagnosis of lung cancer established through histopathological, radiological, or multidisciplinary clinical evaluation and if sufficient clinical and imaging data were available for analysis. Tuberculosis status was assessed for all eligible patients and categorized into three groups: no TB, post-TB sequelae, and active TB.

Patients with missing essential clinical documentation, uncertain tuberculosis status, duplicate records, or exclusively benign pulmonary pathology were excluded from the study. Cases lacking sufficient radiological or oncological information required for subgroup analyses were excluded only from the corresponding statistical evaluations.

Of the 844 patients included in the study cohort, 759 (89.9%) had a confirmed histopathological diagnosis of lung cancer, comprising adenocarcinoma (*n* = 534, 63.3%), non-small cell lung cancer not otherwise specified (*n* = 97, 11.5%), squamous cell carcinoma (*n* = 84, 10.0%), and small cell carcinoma (*n* = 44, 5.2%). The remaining 85 patients (10.1%) did not have a definitive histopathological subtype confirmed: 30 cases (3.6%) presented atypical or suspicious cytological findings, 26 cases (3.1%) had inconclusive or non-diagnostic biopsy results, and 13 cases (1.5%) received a non-malignant diagnosis on tissue sampling, with the remaining 16 cases lacking a classifiable histopathological result. These 85 patients were retained in the overall descriptive analyses and chi-square association testing, as the primary study aim was to characterize the scar carcinoma phenotype across the full lung cancer cohort regardless of histological subtype confirmation. However, they were excluded from all histological subtype-specific analyses and from the binary logistic regression model to avoid misclassification bias.

The final study cohort consisted of 844 patients included in the descriptive and inferential analyses. All available eligible cases with complete datasets for the variables analysed were incorporated into the corresponding statistical models.

### 2.3. Data Collection

Clinical and radiological information was extracted retrospectively from hospital medical records and electronic databases. Collected variables included demographic characteristics, smoking status, pack-year exposure, oxygen therapy requirement, COPD diagnosis, GOLD stage, respiratory failure, associated comorbidities, histopathological subtype, tumour stage, metastatic status, and respiratory symptomatology.

#### Definition of Post-Tuberculosis Sequelae

Post-tuberculous sequelae were defined by characteristic structural lung abnormalities on thoracic imaging together with a compatible history of prior pulmonary tuberculosis. Diagnostic findings included apical fibrosis, fibronodular lesions, calcified granulomas, traction bronchiectasis, pleural thickening, parenchymal distortion, residual cavities, volume loss, and calcified mediastinal lymph nodes. Thoracic CT was the preferred imaging modality; when unavailable, chest radiographs and medical records were reviewed. Prior microbiological confirmation was documented in 220 patients (26.1%), while previous anti-tuberculous treatment was recorded in 298 (35.3%). Microbiological evidence was not mandatory when typical radiological abnormalities coexisted with a compatible clinical history. To reduce misclassification, patients with only diffuse emphysema, centrilobular nodularity, or lower-lobe-predominant fibrosis lacking TB-specific features were excluded. Owing to the retrospective design, blinded image reassessment and interobserver agreement analyses were not feasible, and some risk of misclassification remains a study limitation.

Previous microbiological confirmation of tuberculosis was considered when available in medical records; however, mandatory microbiological documentation was not required for classification as post-TB sequelae in cases presenting characteristic structural abnormalities associated with compatible clinical history or prior documented tuberculosis diagnosis.

To reduce classification bias, isolated emphysematous changes, smoking-related fibrosis, or nonspecific COPD-associated chronic pulmonary abnormalities were not independently classified as post-TB sequelae unless associated with characteristic post-tuberculous structural lesions. Patients presenting only diffuse smoking-related emphysema or nonspecific chronic interstitial abnormalities without radiological features suggestive of prior tuberculosis were categorized outside the post-TB group.

### 2.4. Statistical Analysis

Statistical analysis was performed using IBM SPSS Statistics version 30.0 (IBM Corp., Armonk, NY, USA). Continuous variables were expressed as mean ± standard deviation or median values with confidence intervals, depending on data distribution. Categorical variables were reported as absolute frequencies and percentages.

Associations between the scar carcinoma phenotype and clinicopathological or radiological variables were evaluated using Pearson’s Chi-square test and Fisher’s exact test, as appropriate. Chi-square analyses were performed to investigate the relationships between scar carcinoma and multiple tuberculosis-associated pulmonary abnormalities, including TB sequelae, cavitary/destructive lesions, fibrotic/interstitial/bronchial changes, atelectatic/retractile abnormalities, infectious/inflammatory pulmonary changes, pulmonary opacities/condensation, and pulmonary tumour or expansive mass formation.

Binary logistic regression analysis was additionally performed to identify independent clinical and radiological factors associated with the scar carcinoma phenotype. Variables for the binary logistic regression model were selected based on biological plausibility, prior clinical evidence, and data completeness. Univariate chi-square screening was also used to identify variables associated with the outcome before multivariable analysis. Age, sex, cancer stage, and tuberculosis status were excluded from the primary model because of overlap with the criteria defining the scar carcinoma phenotype, which could introduce multicollinearity and bias estimates. A sensitivity analysis including these variables as additional covariates yielded similar results, with pulmonary opacities/condensation and haemoptysis remaining significantly associated with the scar carcinoma phenotype. Odds ratios (Exp(B)), regression coefficients, Wald statistics, and *p*-values were calculated for all included predictors.

Receiver operating characteristic (ROC) curve analysis and precision–recall curve analysis were subsequently performed to evaluate the discriminatory and predictive performance of the multivariate logistic regression model. Overall model quality and predictive performance were assessed using the area under the ROC curve (AUC) and graphical predictive analyses. Internal validation of the logistic regression model was performed using bootstrap resampling with 2000 iterations to estimate a bias-corrected confidence interval for the area under the receiver operating characteristic curve (AUC = 0.703, 95% CI: 0.667–0.739). Model calibration was assessed using the Hosmer–Lemeshow goodness-of-fit test. External validation in an independent cohort was not performed, which represents a recognized limitation of the present study and restricts the generalizability of the predictive model beyond the analyzed population.

Graphical exploratory analyses, including ROC curves, precision–recall curves, overall model quality plots, and schematic clinicopathological illustrations, were generated to further evaluate the overlap between post-tuberculous pulmonary remodelling and scar-associated carcinogenesis. A *p*-value < 0.05 was considered statistically significant throughout all analyses.

### 2.5. Ethical Considerations

The study was conducted in accordance with the ethical principles of the Declaration of Helsinki. Ethical approval was obtained from the Ethics Committee of the Clinical Pneumophtisiology Hospital Constanța, Romania (Approval No. 473/11 February 2020). The approval remained valid throughout the entire study interval.

## 3. Results

### 3.1. Population Characteristics

The study population consisted of 844 patients evaluated for demographic, clinical, and laboratory characteristics. Descriptive statistical analysis was performed for continuous variables using mean, standard deviation (SD), median values, and 95% confidence intervals (CI) for the median, while categorical variables were summarized using absolute frequencies and percentages. The analyzed parameters included demographic distribution, smoking status, comorbidities, respiratory function, oxygen therapy requirements, and routine laboratory investigations.

All patients included were diagnosed with LC. They were predominantly male (56.9%), with a mean age of 67 ± 9 years (Table 1). Smoking history was highly prevalent, being documented in 68.2% of patients, with a mean pack-year index of 40 ± 25 years. COPD was present in more than half of the cohort (54.9%). Regarding COPD severity, GOLD II and GOLD III stages accounted for the majority of cases (16.5% and 17.3%, respectively), while GOLD IV disease was identified in 19.2% of patients, indicating a substantial burden of advanced airflow limitation.

Pulmonary functional impairment was common and predominantly characterized by obstructive or mixed ventilatory dysfunction. Obstructive dysfunction was identified in 29.1% of patients, while mixed ventilatory impairment was present in 23.5% of cases.

Advanced respiratory impairment was common, with respiratory failure identified in 42.8% of cases and oxygen therapy required in 40.3% of patients. Mean peripheral oxygen saturation (SpO_2_) was 90 ± 9%, with a median value of 94%, reflecting the significant respiratory compromise observed in the analysed population.

Inflammatory biological markers were generally elevated, particularly ESR, which showed a mean value of 51 ± 32 mm/h, reflecting the chronic inflammatory burden of the cohort. Haematological evaluation demonstrated a mean haemoglobin level of 10 ± 3 g/dL, suggesting frequent anaemia, while leukocyte and platelet counts averaged 10 ± 4 × 10^3^/µL and 324 ± 118 × 10^3^/µL, respectively. Metabolic and biochemical parameters revealed a mean blood glucose level of 117 ± 40 mg/dL, mean creatinine of 1.06 ± 0.49 mg/dL, and mean urea level of 43 ± 24 mg/dL. Liver function tests showed mean AST and ALT values of 29 ± 15 U/L and 23 ± 21 U/L, respectively.

Comorbid cardiovascular and metabolic diseases were also frequent, including heart failure in 30.7% of patients and diabetes mellitus in 29.3%. The demographic distribution revealed a slightly higher proportion of patients from urban areas (53.1%) compared to rural areas (46.4%).

### 3.2. Histopathological, Radiological, and Functional Characteristics

Post-TB sequelae represented the predominant TB-related category, being identified in more than half of the patients (58.2%), whereas active TB was relatively uncommon, accounting for only 7.8% of the cohort (Table 2).

Previous microbiological confirmation of tuberculosis was documented in 220 patients (26.1%), while a prior history of anti-tuberculosis treatment was identified in 298 patients (35.3%). Post-TB sequelae represented the predominant TB-related category, being identified in 58.2% of patients, whereas active TB was relatively uncommon, accounting for only 7.8% of cases.

Adenocarcinoma was the most frequent histopathological subtype, accounting for 63.3% of all diagnoses, followed by NSCLC-NOS (11.5%) and squamous cell carcinoma (10.0%). A descriptive tendency toward coexistence with cavitary, fibrotic, and emphysematous changes was observed; however, formal statistical testing of this specific association was not performed in the present analysis and warrants further investigation. These radiological characteristics are highly suggestive of scar-associated carcinogenesis developing on a background of chronic inflammatory and fibrotic pulmonary injury. Small cell carcinoma accounted for 5.2% of cases, while atypical or suspicious malignant findings were observed in 3.6% of patients.

Most patients presented advanced oncological disease at diagnosis, with stage III and IV tumors accounting for 28.1% and 30.5% of the cohort, respectively. Furthermore, metastatic dissemination was identified in 60.1% of patients, emphasizing the predominance of advanced-stage lung cancer within the analyzed population.

Radiologically, pulmonary tumor or expansive masses were identified in 80.0% of patients, while nodular lesions or metastatic dissemination were present in 50.7% of cases. Fibrotic/interstitial/bronchial abnormalities represented one of the most prevalent imaging findings, being observed in 67.7% of patients, closely followed by cavitary and destructive lesions in 69.0% of cases. Atelectatic and retractile changes were also highly prevalent, affecting 65.4% of the cohort, while emphysematous and chronic obstructive changes were identified in 52.7% of patients. Infectious or inflammatory radiological abnormalities were present in 60.4% of cases, further supporting the coexistence of chronic inflammatory pulmonary damage and malignant disease.

Pulmonary opacities or condensation were identified in 45.0% of patients, whereas mediastinal or hilar adenopathy and pleural involvement were observed in 17.4% and 19.3% of cases, respectively. Overall, the radiological profile highlighted the extensive overlap between chronic TB-related pulmonary destruction and lung cancer, particularly among patients presenting cavitary-fibrotic remodeling patterns suggestive of scar carcinoma development.

### 3.3. Clinical Symptoms and Physical Examination Findings

Clinical presentation and respiratory examination findings were evaluated in all patients included in the study cohort. Respiratory symptoms, constitutional manifestations, auscultatory findings, and symptom severity scales were analyzed using categorical frequency distributions and percentages to characterize the clinical burden associated with pulmonary pathology. Table 3 describes the clinical symptoms and physical examination findings observed in the study population.

Vesicular murmur was preserved in 67.3% of patients, diminished in 23.8%, and absent in 8.9%. Regarding pulmonary auscultation findings, no pathological rales were identified in 44.7% of cases. Crackles represented the most frequent abnormal auscultatory finding, followed by bronchial rales, fine crackles, rhonchi, and wheezing. The predominance of crackles and bronchial rales reflected the extensive structural pulmonary damage associated with chronic post-tuberculous changes and malignant parenchymal involvement, particularly in patients with squamous cell carcinoma and cavitary lesions.

Cough characterization revealed dry cough in 46.8% of patients, while productive cough was documented in 30.1%; only 23.1% reported no cough. Haemoptysis was identified in 31.5% of cases.

Pain-related symptoms were highly prevalent (*n* = 651), while constitutional, infectious, or digestive symptoms were observed in 286 cases. Missing, unclear, or absent major symptoms were recorded in 138 patients.

Dyspnea severity assessment showed no dyspnea in 49.9% (*n* = 421) of patients, mild dyspnea in 25.6% (*n* = 216), moderate dyspnea in 15.8% (*n* = 133), and severe dyspnea in 8.8% (*n* = 74). Chest pain severity was absent in 25.9% (*n* = 219) of patients, mild in 41.4% (*n* = 349), moderate in 25.6% (*n* = 216), and severe in 7.1% (*n* = 60), reflecting the substantial symptomatic burden associated with advanced thoracic malignancy and chronic pulmonary destruction.

### 3.4. Association of the Scar Carcinoma Phenotype with Clinicopathological and Radiological Characteristics: Chi-Square Analysis

Chi-square analyses were performed to evaluate the associations between the scar carcinoma phenotype and multiple clinicopathological and radiological variables (Supplementary Material Tables S1–S7, Figures S1–S7). The investigated parameters included TB sequelae, adenocarcinoma histology, infectious/inflammatory pulmonary changes, atelectatic/retractile abnormalities, fibrotic/interstitial/bronchial remodelling, cavitary/destructive lesions, and pulmonary tumour or expansive mass formation. Table 4 illustrates the major clinicopathological and radiological variables significantly associated with the scar carcinoma phenotype.

Statistically significant associations were identified between the scar carcinoma phenotype and all analyzed variables (*p* < 0.001 for all comparisons). Effect size analysis using the Phi coefficient (φ) and Cramér’s V demonstrated very large effect sizes for all associations except pulmonary tumour/expansive mass formation, which showed a large effect size. The strongest associations were observed for TB sequelae (χ^2^ = 811.850, φ = 0.978, V = 0.978), adenocarcinoma histology (χ^2^ = 655.545, φ = 0.879, V = 0.879), and infectious/inflammatory pulmonary changes (χ^2^ = 635.168, φ = 0.865, V = 0.865). Atelectatic/retractile pulmonary changes (χ^2^ = 597.346, φ = 0.839, V = 0.839), fibrotic/interstitial/bronchial abnormalities (χ^2^ = 539.895, φ = 0.797, V = 0.797), and cavitary/destructive lesions (χ^2^ = 508.347, φ = 0.773, V = 0.773) also demonstrated very large effect sizes. Pulmonary tumour or expansive mass formation showed a lower, though still statistically significant, association (χ^2^ = 282.726, φ = 0.576, V = 0.576), corresponding to a large effect size according to Cohen’s benchmarks (V ≥ 0.5). Fisher’s exact test confirmed statistical significance for all analyzed variables. No expected cell counts were below 5, confirming that the assumptions required for chi-square testing were satisfied.

### 3.5. Multivariate Logistic Regression Analysis of Factors Associated with the Scar Carcinoma Phenotype

Binary logistic regression analysis was performed to evaluate the independent associations between selected clinicopathological and radiological variables and the scar carcinoma phenotype. The multivariate model included hemoptysis, smoking status, COPD, nodular/metastatic dissemination, mediastinal or hilar adenopathy, pleural involvement, emphysematous/chronic obstructive pulmonary changes, and pulmonary opacities or condensation.

Multivariate logistic regression analysis identified haemoptysis (B = −0.429, Wald = 7.946, *p* = 0.005, OR = 0.651, 95% CI: 0.483–0.877) and pulmonary opacities/condensation (B = 0.295, Wald = 4.215, *p* = 0.040, OR = 1.343, 95% CI: 1.013–1.781) as statistically significant independent variables associated with the scar carcinoma phenotype. Haemoptysis demonstrated a negative association with the scar carcinoma phenotype (OR < 1), indicating that its presence is associated with lower odds of this phenotype, consistent with the predominance of endobronchial and centrally located tumour subtypes (e.g., squamous cell carcinoma) in patients presenting haemoptysis. Pulmonary opacities/condensation demonstrated a positive independent association with the scar carcinoma phenotype (OR = 1.343). Emphysematous and chronic obstructive pulmonary changes demonstrated borderline statistical significance (B = 0.257, Wald = 3.264, *p* = 0.071, OR = 1.293, 95% CI: 0.979–1.708). Smoking status, COPD, nodular/metastatic dissemination, mediastinal or hilar adenopathy, and pleural involvement did not demonstrate statistically significant independent associations in the multivariate model (*p* > 0.05 for all comparisons). The regression coefficients, Wald statistics, odds ratios, and 95% confidence intervals for all included variables are presented in Table 5.

### 3.6. ROC Curve Analysis of the Predictive Model for the Scar Carcinoma Phenotype

**Figure 3:**
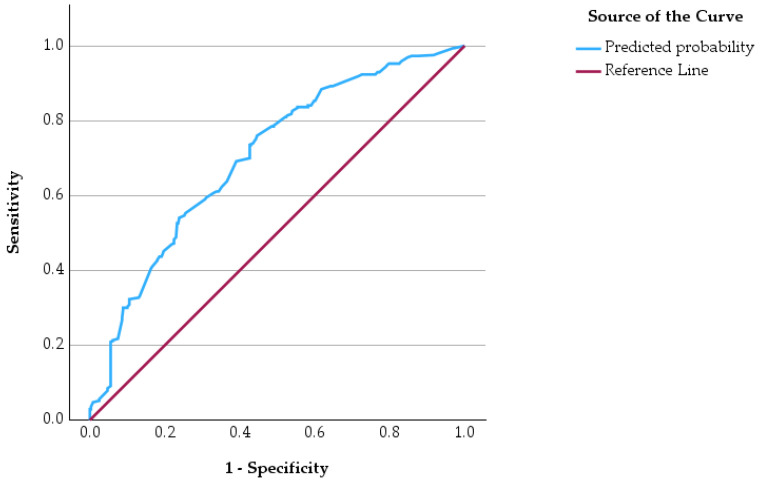
ROC curve analysis of the multivariate logistic regression model for predicting the scar carcinoma phenotype.

Receiver operating characteristic curve analysis was performed to evaluate the discriminatory performance of the multivariate logistic regression model for predicting the scar carcinoma phenotype (Figure 3).

The discriminatory performance of the multivariate logistic regression model was evaluated by receiver operating characteristic curve analysis, yielding an area under the curve of 0.703 (bootstrapped 95% CI: 0.667–0.739, based on 2000 resampling iterations), indicating moderate predictive accuracy for identifying the scar carcinoma phenotype. Model calibration was assessed using the Hosmer–Lemeshow goodness-of-fit test (χ^2^ = 9.509, df = 8, *p* = 0.301), which demonstrated no statistically significant deviation between observed and predicted outcomes, supporting adequate model calibration.

**Figure 4:**
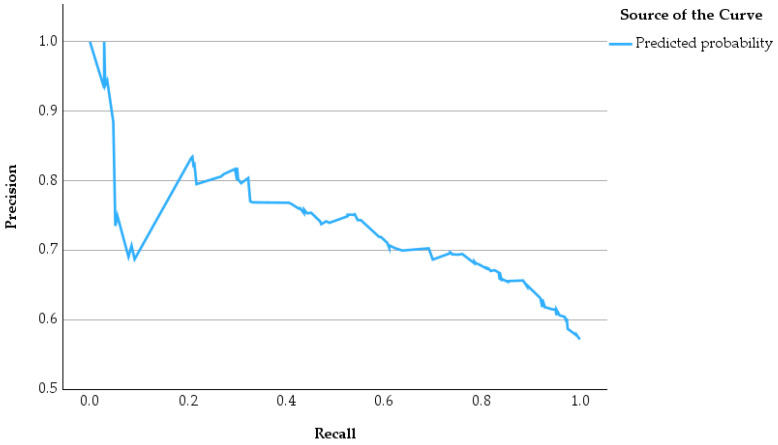
Precision–recall curve analysis of the multivariate logistic regression model for predicting the scar carcinoma phenotype.

The precision–recall curve demonstrated stable predictive performance across a broad range of recall values (Figure 4).

The precision–recall curve demonstrated stable predictive performance across a broad range of recall values, with precision remaining predominantly between 0.70 and 0.80 throughout intermediate recall intervals. External validation of the model in an independent patient cohort was not performed in the present study, which limits the generalizability of these findings and represents a methodological limitation that future multicentre studies should address.

**Figure 5:**
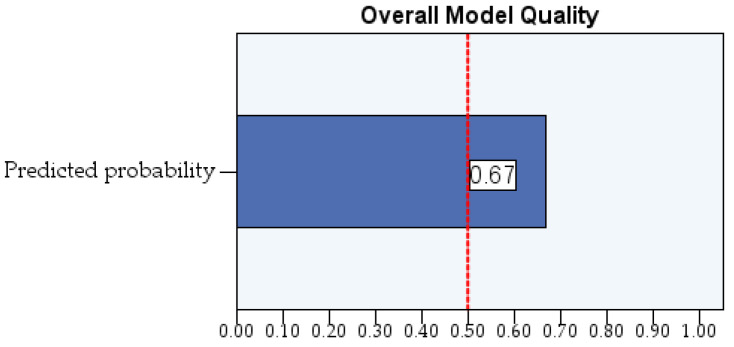
Overall quality assessment of the predictive model for the scar carcinoma phenotype. The model demonstrated an overall predictive quality value of 0.67, indicating predictive performance above the reference threshold of 0.50 and supporting moderate discriminatory capacity for identifying the scar carcinoma phenotype.

The overall model quality analysis demonstrated a predictive performance value of 0.67, exceeding the reference threshold of 0.50 (Figure 5). This finding indicates that the multivariate logistic regression model achieved moderate discriminatory capacity for identifying the scar carcinoma phenotype. The model therefore showed predictive performance superior to random classification based on the analyzed clinicopathological and radiological variables.

## 4. Discussion

Our findings support the growing evidence that post-tuberculous pulmonary sequelae represent an important substrate for the development of lung carcinoma, particularly scar-associated malignancies. In our cohort, the most frequent radiologic abnormalities included fibrotic and retractile changes, cavitary lesions, pleural involvement, and the appearance of expansive pulmonary masses within previously damaged lung parenchyma. These observations are consistent with the review published by Sun et al., who emphasized that chronic inflammation, fibrosis, repeated epithelial injury, and tissue remodeling following pulmonary tuberculosis contribute significantly to carcinogenesis and the development of tuberculosis scar carcinoma [19].

The very high Cramér’s V values for TB sequelae (V = 0.978) and related radiological variables partially reflect the definitional overlap inherent in the composite phenotype definition.

These chi-square analyses are intended for descriptive phenotypic characterization rather than as independent epidemiological associations.

Similarly, Vaishnav and Pailla described pulmonary tuberculosis as an important precursor lesion for subsequent lung cancer, particularly in patients presenting with chronic cavitary disease and residual fibrotic scars [20]. Their report highlighted the diagnostic challenge of differentiating post-TB structural abnormalities from emerging neoplastic lesions, especially when progressive wall thickening, nodularity, or new soft tissue masses develop within old scars [20]. Our results parallel these observations, as several patients demonstrated overlapping fibrotic and destructive changes associated with suspicious tumor-like lesions.

Furthermore, the recent meta-analysis conducted by Gao et al. confirmed a statistically significant association between previous tuberculosis infection and increased lung cancer risk [21]. The authors underlined that persistent inflammatory activity and delayed radiologic surveillance may contribute to late cancer diagnosis in patients with post-TB sequelae [21]. Our study further supports this concept, as many imaging findings initially resembled inactive fibrotic disease or chronic inflammatory changes before malignant progression became evident.

The logistic regression model, based on clinical predictors not used to define the phenotype, provides a more methodologically independent assessment. Thus, the logistic regression model demonstrated moderate discriminatory performance (AUC = 0.703, 95% CI: 0.667–0.739), which, while statistically superior to random classification, does not support standalone clinical use for scar carcinoma phenotype identification. The model should therefore be regarded as a hypothesis-generating tool that highlights potential clinical and radiological correlates of the phenotype rather than a validated predictive instrument. Future prospective studies incorporating histomolecular markers, advanced imaging biomarkers, and standardized CT acquisition protocols will be required to develop models with sufficient discriminatory accuracy for clinical application. External validation in independent cohorts from high-tuberculosis-burden settings is necessary before any clinical translation can be considered.

Here, we reinforce the importance of careful radiologic follow-up in patients with post-tuberculous pulmonary fibrosis, cavitary lesions, and chronic retractile abnormalities. These imaging patterns may conceal or precede the development of scar carcinoma.

The present findings carry important clinical implications, particularly in settings with a high burden of pulmonary tuberculosis. One of the principal diagnostic challenges in this patient population is the radiological differentiation of progressive scar carcinoma from stable post-tuberculous structural changes on computed tomography. A recent study conducted by Zhumagaliyeva et al. takes into consideration the genetic factors as well, mentioning genes such as MUC5B, telomere-related genes, and surfactant protein genes which have provided fundamental insight into pathogenesis of the disease [22]. Kalla et al. also reviewed radiological data from aprox. 900 patients over a 5-year period with a histologically confirmed diagnosis of lung cancer, and describe the presence of scarring within the lungs present on computed tomography of the chest [23]. Their findings demonstrate a clear association, with one-third of the patients reviewed having scar tissue present within the lungs within the same lobe as the diagnosed cancer.

Fibrotic bands, residual cavitary lesions, pleural thickening, and retractile abnormalities are common to both conditions and may obscure or delay the recognition of emerging malignancy [24,25]. In this context, longitudinal computed tomography surveillance is strongly recommended for patients with documented post-tuberculous sequelae, as interval comparison of imaging findings remains the most reliable method for detecting progressive changes [26,27,28]. The appearance of new features should prompt a low-threshold biopsy strategy, given that the window for curative-intent treatment narrows considerably with delayed diagnosis [29,30]. Additionally, the complexity of this radiological overlap brings forward the value of multidisciplinary team evaluation in high-tuberculosis-burden centers, integrating the expertise of pulmonologists, radiologists, thoracic surgeons, and oncologists to ensure timely and accurate diagnostic workup in patients presenting atypical or evolving pulmonary lesions on a background of prior tuberculosis.

## 5. Limitations

This study has several limitations. The study was conducted at a single tertiary center in a high-tuberculosis-burden region of Romania, which could mean that other clinical settings or populations with different tuberculosis epidemiology and lung cancer profiles can have different outcomes. Then, the classification of post-TB sequelae was based on retrospective imaging review without formal blinded reassessment or interobserver agreement analysis, introducing a potential risk of classification bias that cannot be fully quantified. Next, microbiological confirmation of prior tuberculosis was available in only 220 of 844 patients (26.1%), meaning that a proportion of post-TB sequelae classifications relied on radiological and clinical criteria alone.

The logistic regression model was not externally validated in an independent cohort, which restricts the predictive applicability of the model beyond the present dataset. Future prospective, multicentre studies with standardized imaging protocols, systematic microbiological documentation, and external model validation are needed to confirm and extend the present findings.

## 6. Conclusions

In conclusion, our study highlights the complex radiologic spectrum encountered in patients with post-tuberculous pulmonary sequelae and emphasizes the importance of recognizing imaging patterns supporting the hypothesis that it could be malignant transformation. The coexistence of fibrotic, retractile, cavitary, pleural, and expansive pulmonary lesions demonstrates the long-term structural impact of pulmonary tuberculosis on lung parenchyma and underlines the diagnostic difficulty in differentiating chronic post-infectious abnormalities from neoplastic processes.

Among the evaluated imaging findings, fibrotic and interstitial changes, atelectatic and retractile abnormalities, and cavitary lesions were particularly frequent, reflecting the chronic destructive nature of prior tuberculosis infection. At the same time, the identification of pulmonary masses and suspicious nodular components arising within scarred areas supports the hypothesis that post-tuberculous fibrosis may represent a significant substrate for scar carcinoma development. These findings reinforce the concept that chronic inflammation, repeated tissue injury, and abnormal reparative processes may be associated with scar carcinoma phenotype, consistent with the carcinogenesis hypothesis.

Our results also demonstrate that mediastinal involvement, pleural abnormalities, and mixed inflammatory-opacitary changes may coexist with post-TB sequelae, further complicating radiologic interpretation and delaying diagnosis. Consequently, careful longitudinal imaging evaluation remains essential in this patient population, particularly when new lesions, progressive cavity wall thickening, increasing spiculation, or enlarging masses are identified.

## Figures and Tables

**Table 1:** Demographic, clinical, and laboratory characteristics of the study population.

		Mean	SD	Median	95.0% Lower CI for Median	95.0% Upper CI for Median	*N*=	*N* %
Age		67	9	67	67	68		
Gender	Female						364	43.1
	Male						480	56.9
Urban	No						396	46.9
	Yes						448	53.1
Rural	No						452	53.6
	Yes						392	46.4
Hb		10	3	10	10	10		
WBC		10	4	10	10	10		
PLT		324	118	295	290	304		
ESR		51	32	44	41	48		
BLOOD GLUCOSE		117	40	105	102	108		
AST		29	15	27	26	28		
ALT		23	21	21	21	23		
CREATININE		1.06	0.49	0.99	0.97	1.01		
UREA		43	24	35	35	38		
SpO_2_		90	9	94	94	95		
COPD	No						381	45.1
	Yes						463	54.9
GOLD stage	None						381	45.1
	GOLD I						16	1.9
	GOLD II						139	16.5
	GOLD III						146	17.3
	GOLD IV						162	19.2
RESPIRATORY FAILURE	No						483	57.2
	Yes						361	42.8
HEART FAILURE	No						585	69.3
	Yes						259	30.7
Diabetes	No						597	70.7
	Yes						247	29.3
Smoker	No						268	31.8
	Yes						576	68.2
Pack Year Index		40	25	37	35	45		
O2 therapy	No						504	59.7
	Yes						340	40.3
Spirometry	Missing/not performed						215	26.6%
	Normal spirometry						102	12.6%
	Obstructive dysfunction						235	29.1%
	Restrictive dysfunction						39	4.8%
	Mixed dysfunction						190	23.5%
	Non-specific/mild ventilatory impairment						27	3.3%

**Table 2:** Histopathological, radiological, and functional characteristics of the study population.

		*N*=	*N* %
TB status	No TB	287	34.0
	Post TB sequelae	491	58.2
	Active TB	66	7.8
Previous microbiological confirmation of TB	No	624	73.9
	Yes	220	26.1
Previous anti-TB treatment history	No	546	64.7
	Yes	298	35.3
Histopathological diagnosis	Squamous cell carcinoma	84	10.0%
	Small cell carcinoma	44	5.2%
	Undifferentiated carcinoma/lymphoma suspicion	13	1.5%
	Inconclusive/biopsy in progress/no HP confirmation	26	3.1%
	NSCLC, unspecified	97	11.5%
	Adenocarcinoma	534	63.3%
	Other non-malignant diagnosis	6	0.7%
	Mesothelioma/carcinoma–mesothelioma suspicion	6	0.7%
	Pleural sample, no clear malignancy (IHC needed)	4	0.5%
	Atypical cells/suspicious	30	3.6%
Cancer stage	stage I	117	13.9
	stage II	233	27.6
	stage III	237	28.1
	stage IV	257	30.5
Metastases	No	337	39.9
	Yes	507	60.1
Pulmonary tumour/expansive mass	No	169	20.0
	Yes	675	80.0
Nodular lesions/metastatic dissemination	No	416	49.3
	Yes	428	50.7
Mediastinal/hilar involvement and adenopathy	No	697	82.6
	Yes	147	17.4
Pleural involvement	No	681	80.7
	Yes	163	19.3
Atelectatic and retractile changes	No	292	34.6
	Yes	552	65.4
Emphysematous and chronic obstructive changes	No	399	47.3
	Yes	445	52.7
Fibrotic/interstitial/bronchial changes	No	273	32.3
	Yes	571	67.7
Cavitary and destructive lesions	No	262	31.0
	Yes	582	69.0
Pulmonary opacities/condensation	No	464	55.0
	Yes	380	45.0
Infectious/inflammatory changes	No	334	39.6
	Yes	510	60.4

**Table 3:** Clinical symptoms and physical examination characteristics of the study population.

		*N*=	*N* %
Vesicular murmur	present	568	67.3
	diminished	201	23.8
	absent	75	8.9
Rales	none	377	44.7
	bronchial rales	104	12.3
	rhonchi	59	7.0
	crackles	226	26.8
	wheezing	12	1.4
	fine crackles	66	7.8
Pain symptoms	No	193	
	Yes	651	
Constitutional/infectious/digestive symptoms	No	558	
	Yes	286	
Missing/unclear/no major symptoms	No	706	
	Yes	138	
Dyspnea	none	421	49.9
	mild	216	25.6
	moderate	133	15.8
	severe	74	8.8
Chest pain	none	219	25.9
	mild	349	41.4
	moderate	216	25.6
	severe	60	7.1
Cough	none	195	23.1
	dry	395	46.8
	productive	254	30.1
Haemoptysis	no	578	68.5
	yes	266	31.5

**Table 4:** Associations between the scar carcinoma phenotype and major clinicopathological and radiological abnormalities.

Variable Associated with Scar Carcinoma Phenotype	Pearson χ^2^	df	*p*-Value	Fisher’s Exact Test (2-Sided)	Phi Coefficient (φ)	Cramér’s V	Interpretation
TB sequelae	811.85	1	<0.001	<0.001	0.978	0.978	Extremely strong association
Adenocarcinoma histology	655.545	1	<0.001	<0.001	0.879	0.879	Extremely strong association
Cavitary/destructive pulmonary lesions	508.347	1	<0.001	<0.001	0.865	0.865	Very strong association
Atelectatic/retractile pulmonary changes	597.346	1	<0.001	<0.001	0.839	0.839	Very strong association
Fibrotic/interstitial/bronchial changes	539.895	1	<0.001	<0.001	0.797	0.797	Very strong association
Pulmonary tumour/expansive mass formation	282.726	1	<0.001	<0.001	0.773	0.773	Strong association
Infectious/inflammatory pulmonary changes	635.168	1	<0.001	<0.001	0.576	0.576	Extremely strong association

**Table 5:** Multivariate logistic regression analysis of factors associated with the scar carcinoma phenotype.

		B	S.E.	Wald	df	Sig.	Exp(B)	95% CI for Exp(B)
Step 1 ^a^	Patients with haemoptysis vs. patients without haemoptysis	−0.429	0.152	7.946	1	0.005	0.651	0.483–0.877
	Smokers vs. non-smokers	−0.084	0.156	0.289	1	0.591	0.919	0.677–1.248
	Patients with COPD vs. patients without COPD	−0.193	0.146	1.761	1	0.185	0.824	0.619–1.098
	Patients with nodular/metastatic lesions vs. those without them	0.113	0.143	0.617	1	0.432	1.119	0.846–1.482
	Patients with mediastinal/hilar adenopathy vs. those without	−0.278	0.187	2.208	1	0.137	0.757	0.525–1.093
	Patients with pleural involvement vs. those without	−0.066	0.180	0.133	1	0.716	0.936	0.658–1.332
	Patients with emphysematous/chronic obstructive changes vs. those without	0.257	0.142	3.264	1	0.071	1.293	0.979–1.708
	Patients with pulmonary opacities/condensation vs. those without	0.295	0.144	4.215	1	0.040	1.343	1.013–1.781
	Constant	0.333	0.201	2.740	1	0.098	1.395	0.941–2.069

^a^. Variable(s) entered on step 1: Haemoptysis, Smoker, COPD, Nodular lesions/metastatic dissemination, Mediastinal/hilar involvement and adenopathy, Pleural involvement, Emphysematous and chronic obstructive changes, Pulmonary opacities/condensation.

## Data Availability

Data is available on request to the corresponding author.

## Supplementary Materials

The following supporting information can be downloaded at https://www.mdpi.com/article/10.3390/cancers18121935/s1. List of supplementary tables: Table S1. Association between scar carcinoma phenotype and cavitary/destructive pulmonary lesions. Table S2. Association between scar carcinoma phenotype and atelectatic/retractile pulmonary changes. Table S3. Association between scar carcinoma phenotype and fibrotic/interstitial/bronchial pulmonary changes. Table S4. Association between scar carcinoma phenotype and pulmonary tumor/expansive mass formation. Table S5. Association between scar carcinoma phenotype and infectious/inflammatory pulmonary changes. Table S6. Association between scar carcinoma phenotype and TB sequelae. Table S7. Association between adenocarcinoma phenotype and TB sequelae. List of supplementary figures: Figure S1. Distribution of cavitary/destructive pulmonary lesions according to scar carcinoma phenotype. Figure S2. Distribution of atelectatic/retractile pulmonary changes according to scar carcinoma phenotype. Figure S3. Distribution of fibrotic/interstitial/bronchial pulmonary changes according to scar carcinoma phenotype. Figure S4. Distribution of pulmonary tumor/expansive mass formation according to scar carcinoma phenotype. Figure S5. Distribution of infectious/inflammatory pulmonary changes according to scar carcinoma phenotype. Figure S6. Distribution of TB sequelae according to scar carcinoma phenotype. Figure S7. Distribution of adenocarcinoma according to scar carcinoma phenotype.
