# Supplementary material for: Tuberculosis-Associated Scar Carcinoma in Lung Cancer: Clinicopathological and Radiological Features of a Fibrotic-Cavitary Phenotype in a Retrospective Observational Cohort
Source: Cancers (Basel). 2026 Jun 14;18(12):1935. doi: 10.3390/cancers18121935 (PMC13296693; doi:10.3390/cancers18121935)
Supplement: Supplementary file 1 [file cancers-18-01935-s001.zip › cancers-4357480-supplementary.pdf]

SUPPLEMENTARY MATERIAL

Chi-Square Tests

Table S1. Association between scar carcinoma phenotype and cavitary/destructive pulmonary lesions

	Value	df	Asymptotic Significance (2-sided)	Exact Sig. (2-sided)	Exact Sig. (1-sided)
Pearson Chi-Square	508.347[a]	1	<.001		
Continuity Correction[b]	504.963	1	<.001		
Likelihood Ratio	621.494	1	<.001		
Fisher's Exact Test				<.001	<.001
Linear-by-Linear Association	507.745	1	<.001		
N of Valid Cases	844				

a. 0 cells (0.0%) have expected count less than 5. The minimum expected count is 112.06.
b. Computed only for a 2x2 table

Figure S1. Distribution of atelectatic/retractile pulmonary changes according to scar carcinoma phenotype

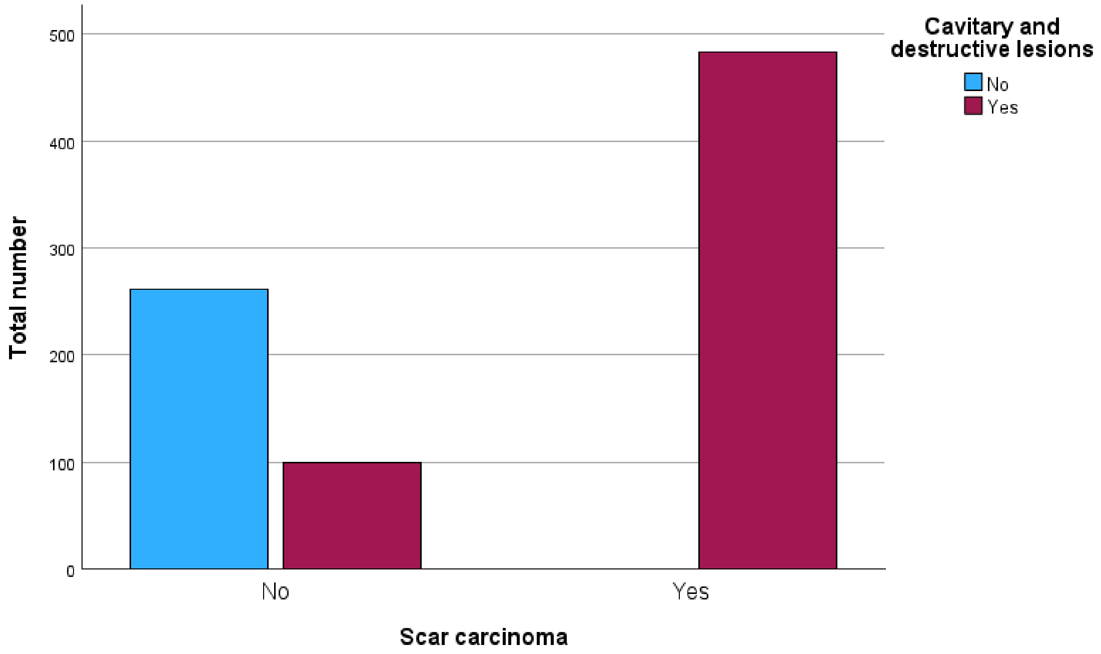

Table S2. Association between scar carcinoma phenotype and atelectatic/retractile pulmonary changes

	Value	df	Asymptotic Significance (2-sided)	Exact Sig. (2-sided)	Exact Sig. (1-sided)
Pearson Chi-Square	597.346[a]	1	<.001		
Continuity Correction[b]	593.777	1	<.001		
Likelihood Ratio	736.381	1	<.001		
Fisher's Exact Test				<.001	<.001
Linear-by-Linear Association	596.639	1	<.001		
N of Valid Cases	844				

a. 0 cells (0.0%) have expected count less than 5. The minimum expected count is 124.90.

b. Computed only for a 2x2 table

Figure S2. Distribution of atelectatic/retractile pulmonary changes according to scar carcinoma phenotype

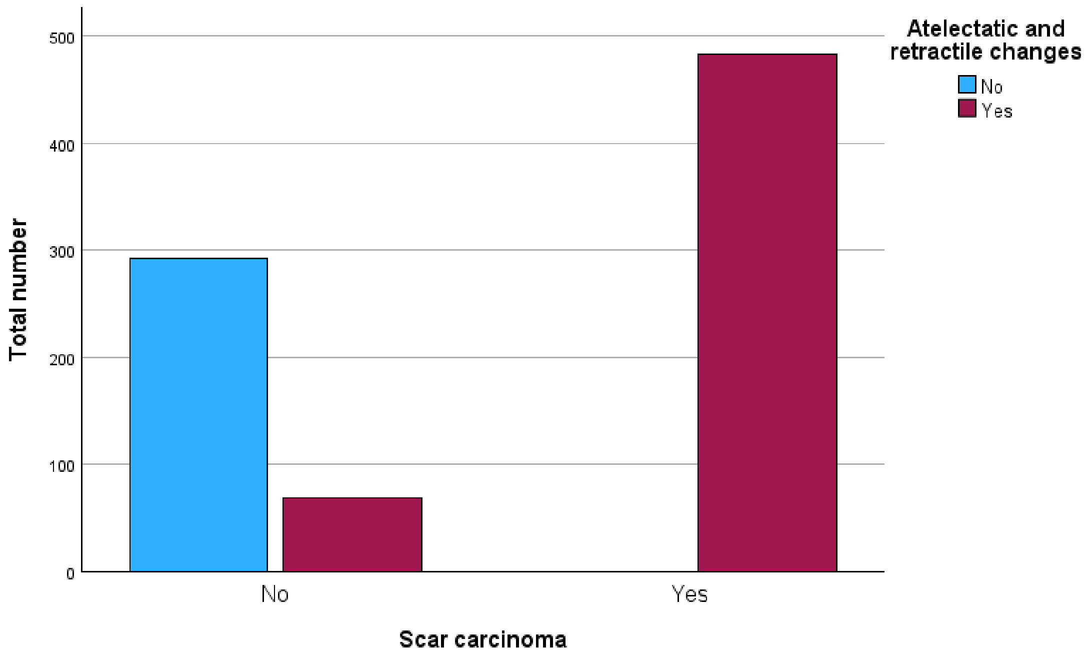

Table S3. Association between scar carcinoma phenotype and fibrotic/interstitial/bronchial pulmonary changes

	Value	df	Asymptotic Significance (2-sided)	Exact Sig. (2-sided)	Exact Sig. (1-sided)
Pearson Chi-Square	539.895[a]	1	<.001		
Continuity Correction[b]	536.444	1	<.001		
Likelihood Ratio	661.524	1	<.001		
Fisher's Exact Test				<.001	<.001
Linear-by-Linear Association	539.255	1	<.001		
N of Valid Cases	844				

a. 0 cells (0.0%) have expected count less than 5. The minimum expected count is 116.77.
b. Computed only for a 2x2 table

Figure S3. Distribution of fibrotic/interstitial/bronchial pulmonary changes according to scar carcinoma phenotype

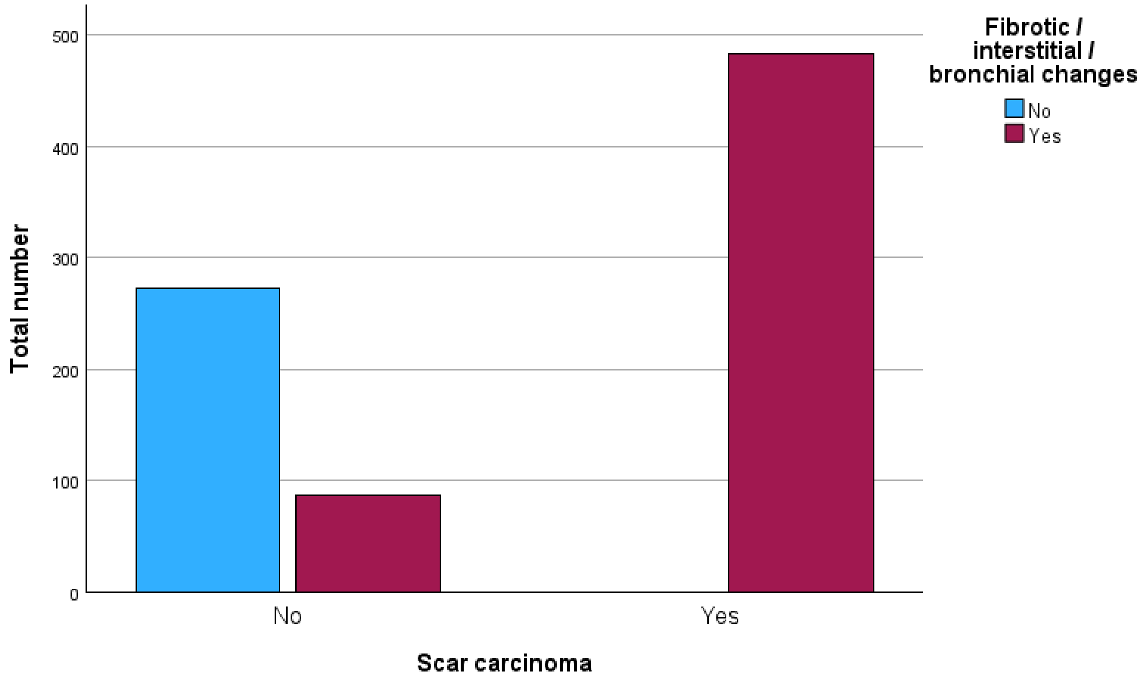

Table S4. Association between scar carcinoma phenotype and pulmonary tumor/expansive mass formation

	Value	df	Asymptotic Significance (2-sided)	Exact Sig. (2-sided)	Exact Sig. (1-sided)
Pearson Chi-Square	282.726[a]	1	<.001		
Continuity Correction[b]	279.810	1	<.001		
Likelihood Ratio	346.248	1	<.001		
Fisher's Exact Test				<.001	<.001

Linear-by-Linear Association	282.391	1	<.001		
N of Valid Cases	844				

a. 0 cells (0.0%) have expected count less than 5. The minimum expected count is 72.29.
b. Computed only for a 2x2 table

Figure S4. Distribution of pulmonary tumor/expansive mass formation according to scar carcinoma phenotype

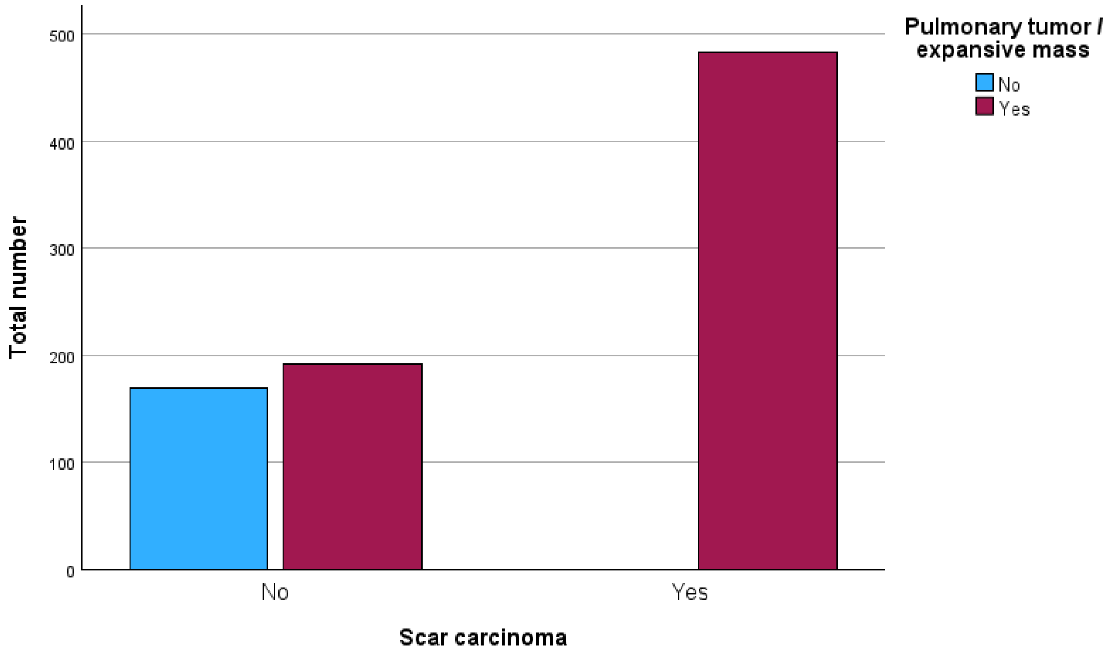

Table S5. Association between scar carcinoma phenotype and infectious/inflammatory pulmonary changes

	Value	df	Asymptotic Significance (2-sided)	Exact Sig. (2-sided)	Exact Sig. (1-sided)
Pearson Chi-Square	635.168[a]	1	<.001		
Continuity Correction[b]	631.588	1	<.001		
Likelihood Ratio	750.792	1	<.001		
Fisher's Exact Test				<.001	<.001
Linear-by-Linear Association	634.416	1	<.001		
N of Valid Cases	844				

a. 0 cells (0.0%) have expected count less than 5. The minimum expected count is 142.86.
b. Computed only for a 2x2 table

Figure S5. Distribution of infectious/inflammatory pulmonary changes according to scar carcinoma phenotype

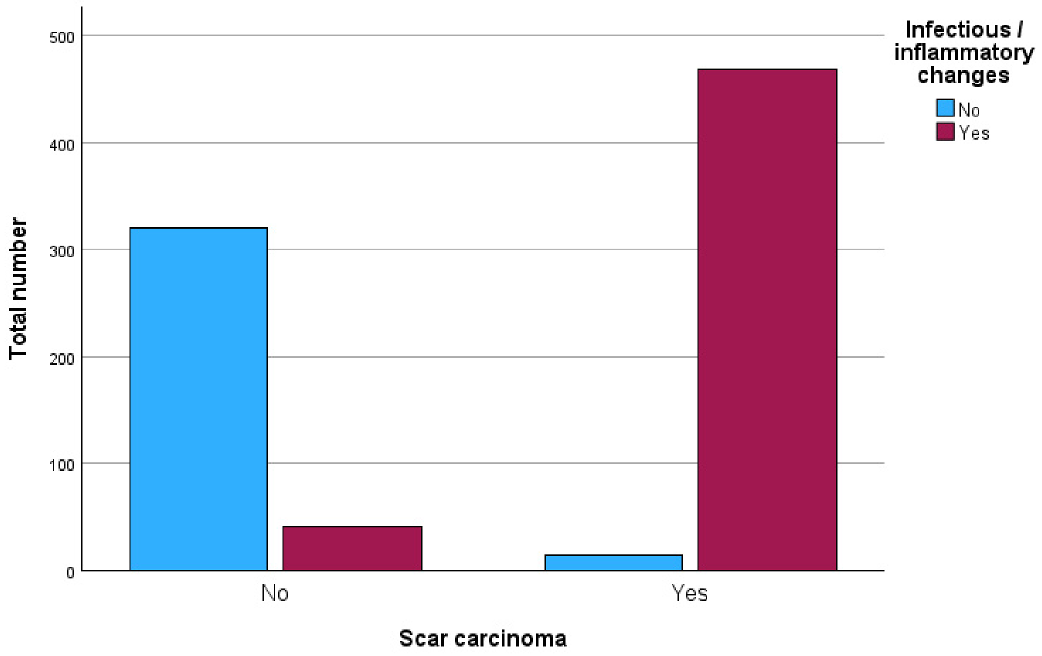

Table S6. Association between scar carcinoma phenotype and TB sequelae

	Value	df	Asymptotic Significance (2-sided)	Exact Sig. (2-sided)	Exact Sig. (1-sided)
Pearson Chi-Square	811.850[a]	1	<.001		
Continuity Correction[b]	807.836	1	<.001		
Likelihood Ratio	1070.594	1	<.001		
Fisher's Exact Test				<.001	<.001
Linear-by-Linear Association	810.888	1	<.001		
N of Valid Cases	844				

a. 0 cells (0.0%) have expected count less than 5. The minimum expected count is 150.99.
b. Computed only for a 2x2 table

Figure S6. Distribution of TB sequelae according to scar carcinoma phenotype

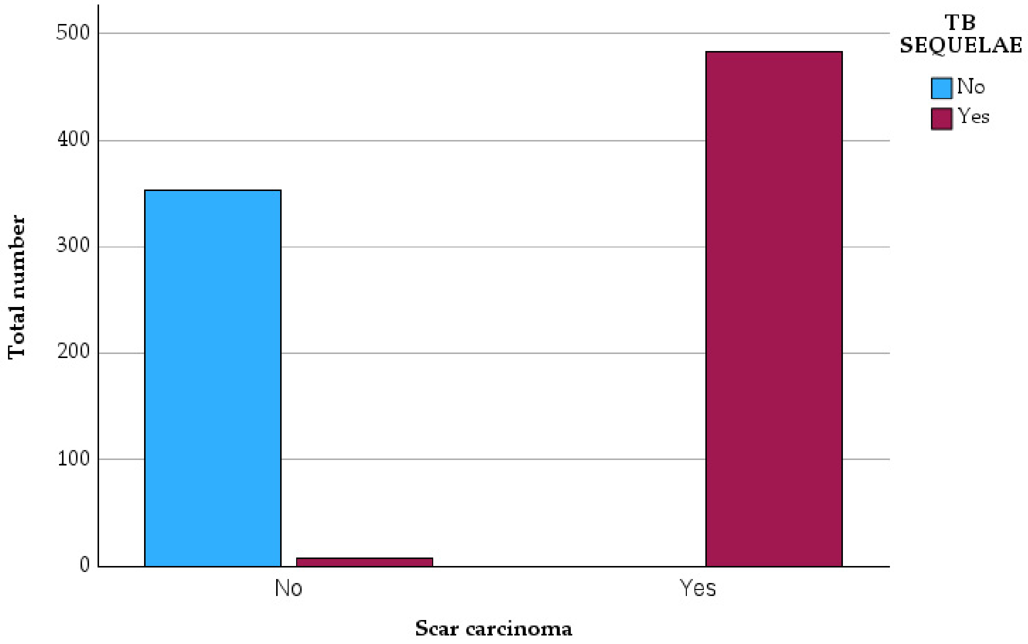

Table S7. Association between adenocarcinoma phenotype and TB sequelae

	Value	df	Asymptotic Significance (2-sided)	Exact Sig. (2-sided)	Exact Sig. (1-sided)
Pearson Chi-Square	655.545[a]	1	<.001		
Continuity Correction[b]	651.855	1	<.001		
Likelihood Ratio	815.815	1	<.001		
Fisher's Exact Test				<.001	<.001
Linear-by-Linear Association	654.769	1	<.001		
N of Valid Cases	844				

a. 0 cells (0.0%) have expected count less than 5. The minimum expected count is 132.59.

b. Computed only for a 2x2 table

Figure S7. Distribution of adenocarcinoma according to scar carcinoma phenotype

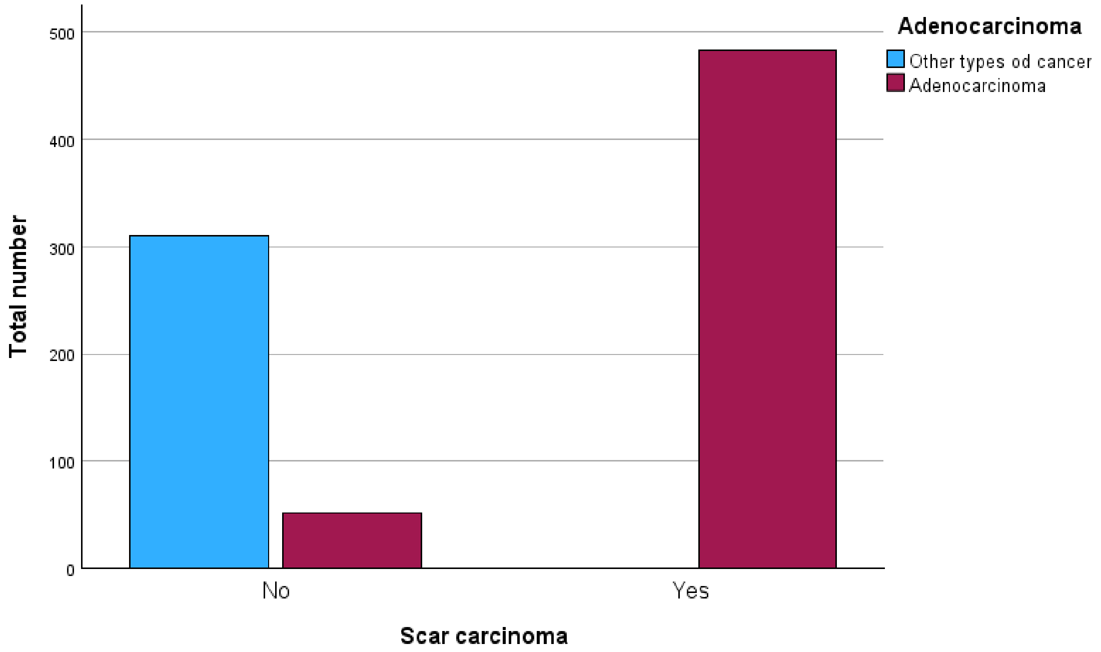

Adenocarcinoma
Other types od cancer
Adenocarcinoma
Total number
500
400
300
200
100
0
No
Yes
Scar carcinoma
